# Electrophysiological and Neurochemical Considerations of Distinct Neuronal Populations in the Rat Pedunculopontine Nucleus and Their Responsiveness Following 6-Hydroxydopamine Lesions

**DOI:** 10.3389/fnins.2019.01034

**Published:** 2019-09-26

**Authors:** Xuenan Wang, Xiwen Geng, Min Li, Jinlu Xie, Dadian Chen, Hongyu Han, Xiaoqian Meng, Xiaomeng Yao, Haiyan Zhang, Yunfeng Gao, Hongli Chang, Xiao Zhang, Yanan Wang, Min Wang

**Affiliations:** ^1^Key Laboratory of Animal Resistance of Shandong Province, College of Life Science, Shandong Normal University, Jinan, China; ^2^Experimental Center, Shandong University of Traditional Chinese Medicine, Jinan, China; ^3^Advanced Material Genome Innovation Team, Advanced Materials Institute, Shandong Academy of Sciences, Qilu University of Technology, Jinan, China; ^4^Department of Physiology, School of Medical Huzhou University, Huzhou Central Hospital, Huzhou, China; ^5^School of Nursing, Qilu Institute of Technology, Jinan, China

**Keywords:** pedunculopontine nucleus, Parkinson’s disease, firing pattern, synchronization, morphology

## Abstract

The pedunculopontine nucleus (PPN) is composed of a morphologically and neurochemically heterogeneous population of neurons, which is severely affected by Parkinson’s disease (PD). However, the role of each subtype of neurons within the PPN in the pathophysiology of PD has not been completely elucidated. In this study, we present the discharge profiles of three classified subtypes of PPN neurons and their alterations after 6-hydroxydopamine (6-OHDA) lesion. Following 6-OHDA lesion, the spike timing of the Type II (GABAergic) and Type III (glutamatergic) neurons had phase-lock with the oscillations in the delta and beta band frequency range in the PPN, respectively. Morphological evidence has shown distinct alteration in three kinds of neurons after 6-OHDA lesion. These findings revealed that the changes in the firing characteristics of neurons in PPN in hemi-parkinsonism rats are closely associated with damaged neuronal morphology, which would make contributions to the divergence of dysfunctions in Parkinsonism.

## Introduction

Parkinson’s disease (PD) results from the progressive loss of the dopaminergic neurons in the substantia nigra (SN) (Muthusamy et al., 2007; Garcia-Ruiz and Espay, 2017), and is characterized by tremor at rest, rigidity, slowness or absence of voluntary movement and freezing (Alam et al., 2012; Janzen et al., 2012). The mainstay of PD treatment is dopamine (DA) replacement with levodopa, which alleviates some of the motor symptoms. However, some side effects, such as dyskinesia, have increasingly emerged, and the gait disturbances respond poorly to this treatment. Recently, low-frequency deep brain stimulation of the pedunculopontine nucleus (PPN) is emerging as an alternative treatment to improve the postural and gait disorders in clinical studies (Alam et al., 2012; Thevathasan et al., 2012).

The PPN is located in the midbrain and is considered a key part of the mesencephalic locomoter region, having an important role in the control of gait (Alam et al., 2011; Petzold et al., 2015). In addition, the PPN interconnects with the basal ganglia, receives a direct input from the cerebral cortex, and provides outputs to the thalamus and spinal cord (Jenkinson et al., 2009; Gut and Winn, 2016). In parkinsonism induced by 6-hydroxydopamine (6-OHDA) lesion, the disruption of the basal ganglia causes dysfunction of the PPN neurons (Wang et al., 2005). However, there is still lack of observation concentrating on the alteration of specific PPN neuron subtypes in this region following a 6-OHDA lesion.

The spike patterns vary after a 6-OHDA lesion, which can generate unique activity of local field potential (LFP). Subsequently, the coherence of the spikes and LFPs becomes a powerful tool to encode neuronal activities. However, it has only been applied for the identification of the spike-LFP relationship in anesthetized rats, which could not reflect actual responsiveness (Walters et al., 2007). There is a critical need to determine the synchronization between spiking activity of individual PPN neurons and the LFP of neural ensembles in freely moving PD animals.

In addition, the PPN is composed of different neuronal populations, such as cholinergic, γ-aminobutyric acid (GABA)-ergic, and glutamatergic neurons (Martinez-Gonzalez et al., 2011; Pienaar and Van, 2013). These neuron types arborize widely and have reciprocal connections with basal ganglia. Based on these observations, several studies are likely to attribute the alteration of the firing pattern to the dysfunction of the cortico-basal ganglia loop in a rat model of PD (Geng et al., 2016b; Li et al., 2016). However, the link between electrophysiological characteristics and neuronal morphology has not been found yet.

To investigate these problems, we recorded and classified the spontaneous discharge of individual neurons in the PPN in control and rat models of PD. Then, we analyzed the spike-LFP coherence and the morphological changes in the PPN following a 6-OHDA lesion. In this manner, the electrophysiological impairments could be related to the morphological alterations in the PPN in the pathophysiology of PD.

## Materials and Methods

### Animals

Adult male Wistar rats, weighing 280–320g (Shandong University, China), were used for the experiments. All rats were housed in a temperature-controlled environment (26°C) with a 12-hour dark/light cycle. Water was available *ad libitum* throughout the experiment and food was limited to 10–20 g/day to maintain a constant animal weight. All animal procedures were conducted in accordance with the National Institutes of Health Guidelines for the Care and Use of Laboratory Animals. The principles for the care and use of laboratory animals in research were strictly followed and all protocols were reviewed and approved by the Shandong Normal University Ethics Review Board. The rats were divided into the following groups: (1) the 6-OHDA lesion group, in which the rats received a unilateral injection of 6-OHDA in the left medial forebrain bundle (MFB); this group was further divided into two subgroups: subgroup 1, wherein the rats were used for electrophysiological analysis, and subgroup 2, wherein the rats were used for the morphological analysis, and (2) the control group, in which the rats received the same volume of vehicle injection (0.02% ascorbic acid in physiological saline) in the left MFB.

### Unilateral 6-OHDA Lesion and Behavioral Assessment

The rats were fully anesthetized with urethane (1.0 g/kg, intraperitoneal injection), and were fixed on a stereotaxic frame. The head was balanced and the skull was exposed by removing the subcutaneous connective tissues. The stereotaxic coordinates for the left MFB (2.16 mm posterior to bregma, 2.10 mm lateral to bregma) were derived from the atlas of rat brain provided by Paxinos and Watson (2007). A small hole was drilled and the overlying dura mater was removed. Prior to the delivery, the 6-OHDA (Sigma, United States) was dissolved in 0.02% ascorbate saline at a concentration of 4 μg/μl (Geng et al., 2016a) A 3 μl solution taken by microsyringe was lowered to the target site at a speed of 1 mm/min: injection site 1 (1.5 μl): dorsoventral: −8.40 mm and injection site 2 (1.5 μl): dorsoventral: −8.65 mm. After the infusion, the microsyringe stayed *in situ* for another 10 min before being slowly withdrawn to allow the solution to diffuse completely. Finally, the incision was sutured and the animal was allowed to recover.

After 2 weeks, the extent of the lesion was tested by injecting apomorphine (0.05 mg/kg, dissolved in 1 ml of 0.9% NaCl solution) into the neck muscle. Only rats that turned consistently from the lesioned side to the non-lesioned side with over seven rotations per minute or 210 rotations within 30 min (Wang et al., 2015) were regarded as successful model of PD. Finally, 68 successful 6-OHDA lesioned rats were used for subsequent experiments. Among them, 60 rats had an electrode implanted, and the rest were used for anatomy experiments.

### Implantation of the Recording Electrode

The electrode consisted of eight nickel-chromium Teflon-insulated microwires (25 μm in diameter, California Fine Wire, Grover Beach, CA, United States) and two silver wires (200 μm in diameter) which served as ground and reference wires. The insulation at one end of the microwires was removed about 2 mm. Then the ends of the microwires were wrapped around the pin of a socket, and stabilized with metallic glues. The other end was gathered into a bundle.

The electrode was implanted into the 60 lesioned rats and 35 control rats. The coordinates for the left PPN is 7.8 mm posterior to bregma, 2.0 mm lateral to bregma and 7.0 mm ventral from the surface of the skull. The electrodes were lowered into the target slowly. Five stainless-steel screws were used to connect the ground and reference wires and fix the electrode with dental cement. After the surgery, the rats were injected with carprofen (5 mg/kg, intraperitoneal injection) and monitored for recovery.

### Electrophysiological Data Acquisition

One week after the implantation, the extracellular activity of the PPN neurons in the control and lesioned groups was recorded using the OmniPlex D Neural Data Acquisition System (Plexon, Inc., Dallas, United States) via a cable. A commutator was used to protect the cable from twisting. The cable was connected to the electrode through the socket. The recording procedure was carried out continuously for 10 min every day, for 1 week. During the recording, the rats were under quiet wakefulness (when they were immobile and inattentive to their environment). The signal was passed through a headstage and the spike and LFP components were extracted in parallel. The spikes and LFP were collected at a sampling rate of 20 and 1000 Hz, and band-pass filtering value was 300–8000 and 0.5–200 Hz, respectively. During the recording sessions, signals were also amplified (2000×) and the activity on the earth wire was seen as the reference. The signal was saved for further analyses using the Offline Sorter V4 (Plexon,Inc., Dallas, United States) and NeuroExplorer V5 (Nex Technologies, United States).

### Identification of the Electrode Position

**FIGURE 1:**
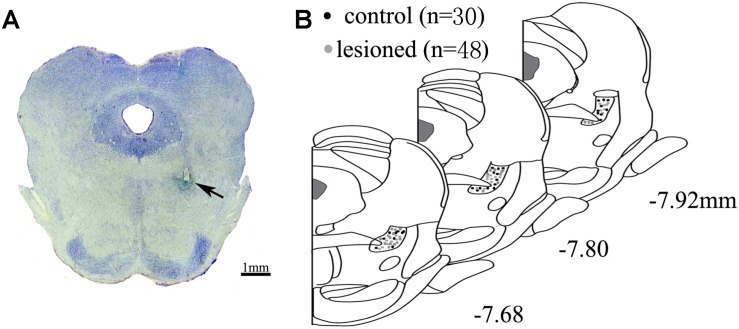
Location of electrode. **(A)** Electrode tips (arrows) were visualized within PPN. Scale bar: 1 mm **(B)** All the placements of electrode tips in control rats (black circle) and lesioned rats (gray circle) are shown according to the distance from bregma in the anteroposterior plane.

After completing the recordings, the rats were anesthetized through an overdose of urethane (1.8 g/kg, intraperitoneal injection), and the recording sites were marked by passing a 10 μA current for 5 s, 10 times. The rats were then perfused via ascending aorta with 200 ml of 0.01 M phosphate-buffered saline (PBS), followed by 200 ml of 4% paraformaldehyde (PFA) containing 1% potassium ferricyanide at a rate of 20 ml/min. After the perfusion, the brains was removed and stored in 4% PFA solution overnight. Subsequently, they were immersed in 30% sucrose in phosphate buffer for 2 days and embedded using OCT. Coronal sections of 50 μm through the PPN were cut using a freezing microtome (CRYOSTAR NX50, Thermo Fisher, United States). The sections containing the tracks and tips of the electrodes were mounted on glass slides and stained with cresyl violet. The signals from the correct electrode sites were used for further analyses (Figure 1). Finally, the correct signal was recorded from the 48 lesioned rats and 30 control rats.

### Data Analysis

#### Spike Sorting and Cell Classification

To detect the signals, the spikes with a signal-to-noise ratio of over 2:1 were extracted. To ensure adequate spikes, a 2-min-length signal was randomly selected as one segment, 14 segments in total for one animal. Firstly, according to the K-means clustering algorithm, different types of neurons from the control group were isolated automatically. Secondly, the waveforms that had short interspike intervals (ISIs) (less than 2 ms) and contained invalid noise were removed manually. Thirdly, in a 3-dimensional (3D) space, different waveforms in the same channel were clustered using principle-component analysis (PCA). Finally, neuron classification was based on the analysis of waveform, duration from trough to peak (between the first negative deflection and the peak of the second positive deflection of a spike waveform) (Wang et al., 2015), firing pattern, and ISIs (Boucetta et al., 2014). In a 3D space, L-ratio and Isolation Distance (ID) were calculated to measure two per-unit sort quality metrics using Offline Sorter V4. L-ratio is a measure of the amount of noise near the unit. A low value of L-ratio indicates the unit is well separate and not contaminated by nearby spikes that are not assigned to the unit. ID is a measure of how distant the non-unit spikes are from the spikes in the unit under consideration. Large values indicate a well-separated unit (Schmitzer-Torbert et al., 2005).

To further assess the effects of the 6-OHDA lesion on the firing rates and firing pattern, we calculated the mean firing rate, coefficient of variance (CV), mode of ISI, and asymmetry index in both groups and we made an intergroup comparison. The mean firing rate was defined as the average number of spikes in a second. We generally used it to measure the rate of discharge. The CV values reflected the regularity degree of neuronal discharge. When the CV values were over 1, there were more irregular firing patterns, while the firing patterns tended to be more regular when the CV values were below 1 (Geng et al., 2016b). The mode values resembled the most frequent ISI. The asymmetry index was the ratio of the mode to the mean ISI (Alam et al., 2012).

#### Local Field Potential

The time–frequency spectrogram and assessment of relevant LFP power were calculated in MATLAB 2015a (The Mathworks, United States). The LFP power spectra were computed using Fast Fourier Transform (FFT) analysis. The Welch method was used the, with a sampling rate of 1000, and a Hanning window was applied. The power spectral density plots of the LFPs showed a visualized graphical representation of the various frequencies. For the assessment of the relevant LFP power, the LFP power spectra were analyzed in different bands of delta (1–4 Hz), theta (4–7 Hz), alpha (7–12 Hz), beta (12–30 Hz), and gamma (30–70 Hz).

#### Spike-Field Coherence

The spike-field coherence (SFC) was used to evaluate the timing relationship between spikes and ongoing oscillations (Grasse and Moxon, 2010; Rutishauser et al., 2010). Briefly, the SFC is a frequency-dependent coherence index (Rutishauser et al., 2010). Formally, the SFC was calculated as the ratio of the frequency spectrum of STA (fSTA) to the spike-triggered power (STP), multiplied by 100 [SFC= (fSTA/STP) × 100%], ranging from 0 to 100%. Basically, each spike fired centrally on the ongoing LFP oscillations (±500 ms long) was regarded as one segment. Averaging these LFP segments derived the spike-triggered average (STA) (van der Meer and Redish, 2009). To quantify the fSTA, we used the following multitaper function with the following settings: 3 time-bandwidth, five tapes and 50% window overlap. The STP was normalized by the average of all power spectra of all segments. To ensure sufficient statistical power, only the signals with at least 50 spikes were used.

#### Phase Estimation

To further illustrate how the spiking activity of individual neurons varied in time with respect to the ongoing PPN LFP oscillation, we analyzed the phase relationships between the neuronal spike timing and the LFP activity in specific frequency bands. In the phase analysis, the value of mean angles and mean vector length were computed using the CircStat toolbox (Berens, 2009). Before that, the Rayleigh test was performed and only *p* < 0.05 represents neuron fired locked on the LFP oscillation. The phase of each spike was measured (in radians) ranging from 0° to ± 180° with the phase equal to the peak and trough of the LFP oscillation. The mean vector length ranged between 0 and 1, where 0 corresponded to a discrete relationship, while 1 corresponded to a concentrated distribution of phase relationship. To guarantee the validity of the statistical results, only neurons that fired at least 50 spikes were assessed. A phase plotted rose circular histogram was exhibited for those spikes showing phase-lock in relation to LFP in PPN.

#### Histology

The rats from subgroup 2 were anesthetized through an overdose of 4% chloralhydrate (1.8 g/kg, i.p). They were treated with the same way as described above. Sequential coronal sections (20 and 50 μm in thickness) were cut at the position of 7.68–7.92 mm posterior to the bregma. The 20-μm-thick sections were mounted on gelatin-coated slides and stained with cresyl violet. The 50-μm-thick sections were collected in PBS and stored in PBS for immunofluorescence analysis.

The PPN in 20-μm-thick section was observed under a low magnification objective (4 × air objective lens) with a camera (OPTEC TP DV500 camera) coupled to a microscope (OLYMPUS CX31) and ToupView 3.7 system software (Toup Tek Photonics Co., Hangzhou, China). Then cells were magnified under a high-magnification objective lens (40×).

#### Immunofluorescence Staining

The 50-μm-thick sections were washed with 0.01-M PBS for 5 min three times. The tissues were then blocked in a solution containing 5% normal donkey serum and 0.5% TritonX-100 for 6 h at room temperature. To observe the nuclei, the sections were labeled with a neuron-specific nuclear protein (NeuN) (1:200, Millipore, catalog#MABN140) containing FITC-conjugated (green) fluorescent dye (SANTA, Inc., catalog#sc-2090). Following location of the NeuN-labeled cells, the relevant section was fluorescently dual-immunostained with goat polyclonal anti-choline acetyltransferase (ChAT) antibody (1:200, Millipore, catalog#AB144P) and donkey anti-goat IgG-TRITC (1:200 SANTA,Inc., catalog#sc-3855), mouse monoclonal anti-glutamate decarboxylase (67 kDa isoform,GAD67) antibody (1:500, Millipore, catalog#5406) and anti-mouse IgG-TRITC (1:500, Abcam, catalog#ab6817), and mouse monoclonal anti-vesicular glutamate transporter 2 (VGluT2) antibody (1:500, Millipore, catalog#5504) and anti-mouse IgG-TRITC for identification of the cells as cholinergic, GABAergic, or glutamatergic. Sections that were not incubated with the primary antibody were used as controls for our protocol. The resulting sections were given a final wash in 0.01-M PBS (4 × 10 min) to stop the continuous reaction. The sections were then mounted on gelatin-coated slides, air dried, and dipped in glycerin containing 20 mg/ml 1,4-diazabicyclo [2.2.2] octane (Sigma, United States) and 10% TrisHcl (0.2 mol/L, pH 7.4). Coverslips were applied and fixed with clear nail polish. The finished sections were stored at −4°C and prepared for a fluorescence microscope.

#### Photomicrographic Processing

The immunolabelled PPN sections were displayed with a laser scanning confocal microscope (Leica TCS SPE, Germany). The location of the PPN was found under a bright-field microscopy (20×). Firstly, we captured the image of the NeuN-immunopositive neurons in the lesioned and non-lesioned sides. Secondly, the images for revealing the cholinergic, GABAergic, and glutamatergic neurons were also obtained. Next, they were merged automatically using the software Plex Util and were stored in the LAS AF series 009 (Leica, Germany). Finally, the images were processed using the Adobe Photoshop software (CS 5.0) to adjust the level of brightness and contrast. The number of immunopositive neurons (NeuN, ChAT, GAD67, and VGluT2) that clearly displayed visible nuclei and somatic cells between the lesioned and non-lesioned sides were counted by three people blind to the experiment using Image Pro Plus 6.0. Meanwhile, the counted neurons were further used for measuring the diameter (μm) of the nucleus and the area size (μm^2^) of the somas using the software Image Pro Plus 6.0. Under the same observation conditions, the morphology of the neurons was observed under oil-immersion objective lenses (63×).

#### Juxtacellular Labeling of Individual Neurons

The recording and labeling experiments were performed on anesthetized rats. A representative individual neuron in PPN was labeled with neurobiotin as described by Pinault (1996). Briefly, the rats were anesthetized and fixed (as described above). For recording, a glass micropipette (2 μm tip and 15–25 MΩ) filled with neurobiotin (Nb, 1.5% w/v, Vector Laboratories) in 2-M NaCl solution was lowered into the PPN. The signal was recorded and analyzed. After the recording of the unit, juxtacellular labeling was applied with a positive pulse of 8 nA (duration: 1 s, 50% duty cycle) for 200 ms.

After the recording, the brain was removed and cut at 50-μm thickness. For identification of the Nb-labeled neurons as ChAT, GABAergic, and glutamatergic by immunofluorescence, the sections were stained for Nb using Cy2-conjugated streptavidin (1:1000, Jackson ImmunoResearch Laboratories). Sections containing Nb-stained neurons were then processed for immunofluorescence for identifying ChAT, GABAergic, or glutamatergic positive neurons, as described in the Immunofluorescence staining section.

### Statistical Analysis

For analyzing the loss of neurons between the non-lesioned side and the lesioned side, Student’s *t*-test was performed. In the ISIs analysis, the comparison of the mean firing rates in both groups were made using Student’s *t*-test. The characteristics of the CV, mode of ISI and asymmetry index were analyzed using the Mann–Whitney *U* test. For the LFP activity and spike-LFP analysis, the comparison of the two groups was made using repeated-measures one-way analysis of variance (ANOVA) with Greenhose-Gessier correction. Only there was a difference between two groups, and comparison of different bands was made using Student’s *t*-test. The differences of the mean phase angles of neurons were tested by the Watson–William test for each frequency band. The differences between the mean vector lengths were tested using the Mann–Whitney *U* test for each frequency band. No corrections for multiple comparisons have been applied in the analysis. The significance for all statistical tests was set at *p* < 0.05. Statistical analysis was performed with a software package Sigma Stat (SPSS18, United States). Data were presented as mean ± the standard deviation.

## Results

### Neuronal Classification in PPN

**FIGURE 2:**
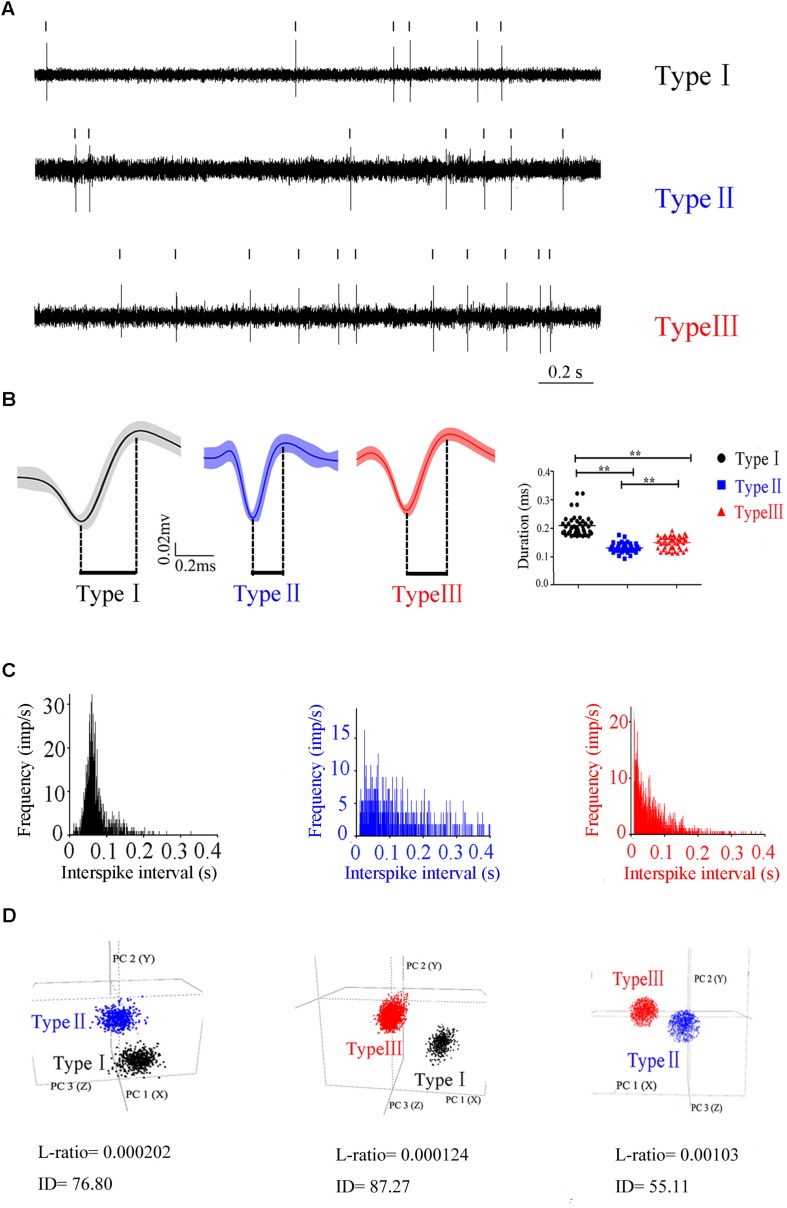
Three different subtypes in PPN identified according to the firing patterns. **(A)** Original signals of the three types of neurons selected for presentation. **(B)** Averaged discharge waveform and spike duration were calculated to identify the difference of three types of neurons.**(C)** ISIs showing the neuronal firing forms. **(D)** Representative separation of different type neurons based on the principle component analysis under 3D view. Type I (black), Type II (blue), Type III (red).

To characterize the neuronal activity of the PPN neurons, *in vivo* recording from the control rats was performed and the signals from 132 neurons were successfully collected. These neurons were classified into three distinct types (Figure 2), based on the trough-to-peak duration of the waveform (Figure 2B) (Weinberger et al., 2008; Pelled et al., 2009; Dupre et al., 2016). We also used PCA to classify waveforms from different single cells (Figure 2D; Fries et al., 2001). Clusters were clearly separated from the origin of the PCA plot. Clusters were regarded as a single unit. The L-ratio and ID value are shown in Figure 2D, and used to evaluate relative high-quality separation (Petrantonakis and Panayiota, 2015). The minimum L-ratio of all clusters was 0.00166, meanwhile, all clusters used in our analyses had ID of at least 50.16. Type I (*n* = 42) had a broad spike duration (0.209 ± 0.035 ms). These neurons discharged at low rates, with a regular firing pattern, and a Poissonian tonic distribution of ISI histogram. Type II (*n* = 40) had a narrow spike duration (0.131 ± 0.012 ms). These neurons had a high firing rate and relatively irregular firing pattern, and the ISI histogram exhibited a sparse distribution. The waveform was in the shape of approximately a “V”. Type III (*n* = 50) had a medium duration of spike (0.154 ± 0.014 ms), longer than that in Type II neuron. These neurons fired fastest of the three, with an irregular firing pattern, and exhibited a concentrated ISI histogram. Out of 132 neurons detected in the control rats, there are 42 Type I, 40 Type II, and 50 Type III neurons.

### Identification, Localization, and Classification of Individual Neuron in the PPN

**FIGURE 3:**
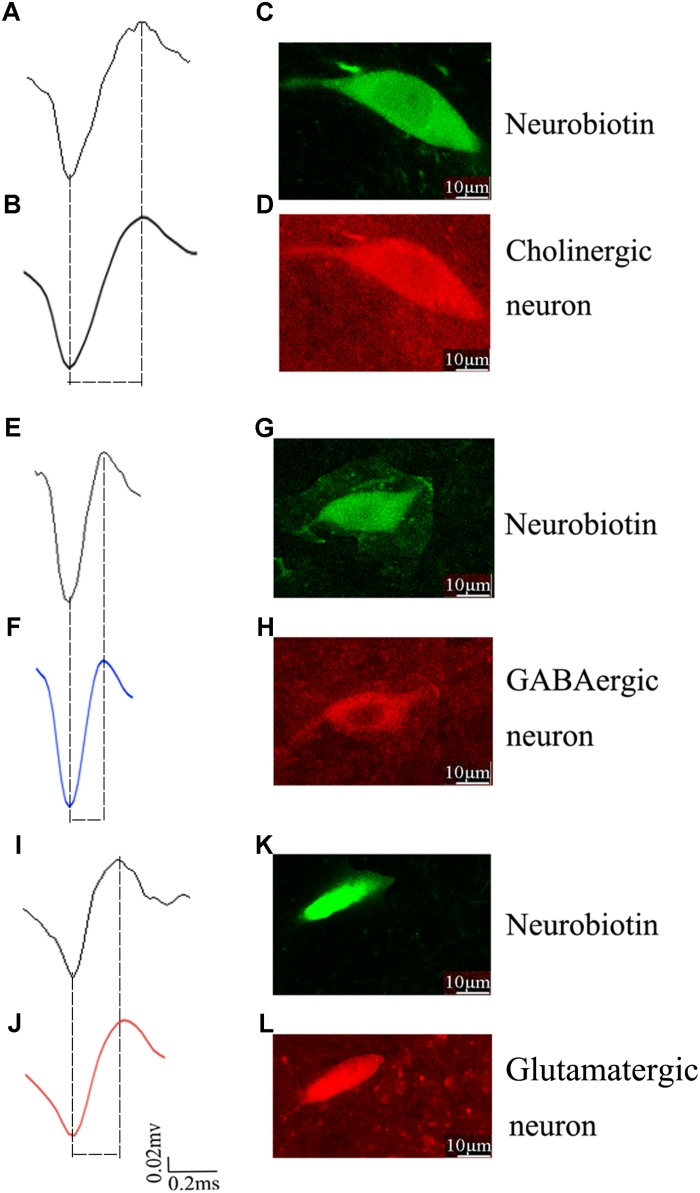
Identification of an individual neuron using the juxtacellular method. A representative extracellular waveform recorded via glass microelectrode **(A,E,I)** and analyzed using Offline Sorter **(B,F,J)** and identified as cholinergic **(C,D)**, GABAergic **(G,H)**, and glutamatergic **(K,L)** by the co-localization of fluorescent markers for the neurobiotin and ChAT, GAD67, VGLuT2.

To identify the molecular markers for these three PPN neuronal types with distinct firing patterns, immunofluorescence staining was performed after the *in vivo* recording (Pesaran et al., 2002). The Type I neurons are positive for choline acetyltransferase (ChAT), a cholinergic marker; the Type II neurons are positive for glutamate decarboxylase (GAD67), a GABAergic marker; and the Type III neurons are positive for vesicular glutamate transporter 2 (VGluT2), a glutamatergic marker (Figure 3).

### Effects of the 6-OHDA-Lesion on the PPN Activity

To study the function of the PPN neurons in the PD model, 6-OHDA was injected unilaterally in the MFB. 48 rats with 6-OHDA lesion surgery were confirmed as PD model by behavioral test and were used for subsequent experiments, and a total of 142 neurons were recorded. Based on the same criteria, they were clustered into three types: 40 Type I, 42 Type II, and 60 Type III.

**FIGURE 4:**
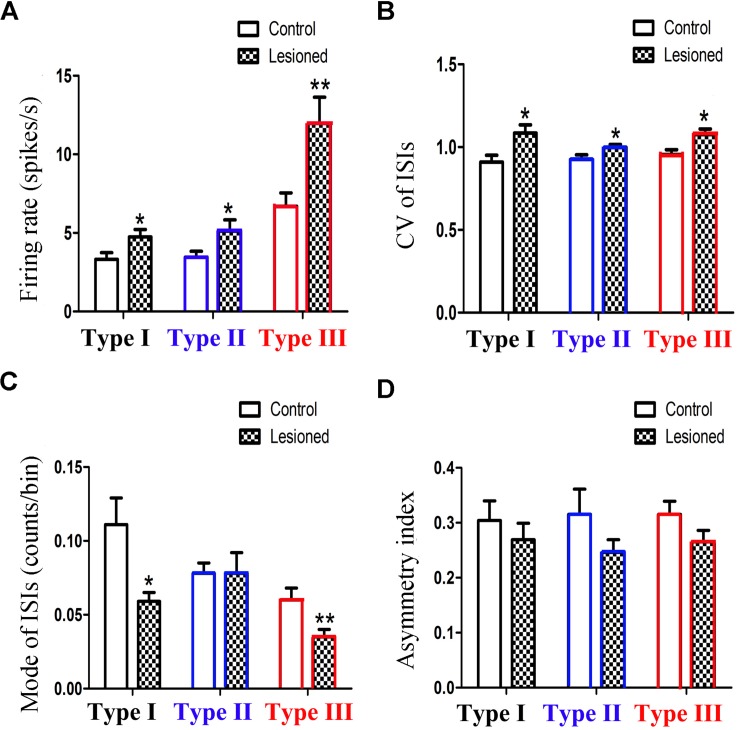
Alteration of the firing rates **(A)**, CV value **(B)**, mode value **(C)** and asymmetry index **(D)** of three subpopulations in PPN after 6-OHDA lesion. (^∗^*p* < 0.05,^∗∗^*p* < 0.01).

To compare the firing property of the neuronal subtypes in the 6-OHDA lesion group with the control group, we found that the firing rates in all three different neuronal subtypes are higher in the 6-OHDA lesion group (Figure 4A): Type I neurons (4.741 ± 0.484 vs. 3.232 ± 0.431, *p* = 0.026; increased by 46.75%), Type II neurons (5.792 ± 0.811 vs. 3.452 ± 0.403, *p* = 0.029; increased by 67.83%), Type III neurons (11.961 ± 1.660 vs. 6.701 ± 0.842, *p* = 0.012, increased by 78.51%).

The CV value (Figure 4B) also showed an increase in the three types of neurons between the control and lesioned groups. For Type I, II, and III neurons, the CV value of the ISIs in the control and lesioned group was 0.909 ± 0.042 vs. 1.084 ± 0.051 (*p* = 0.026), 0.925 ± 0.028 vs. 0.998 ± 0.018 (*p* = 0.032), and 0.955 ± 0.028 vs. 1.076 ± 0.033 (*p* = 0.024), respectively.

As shown in Figure 4C, the 6-OHDA lesion caused a decrease in the mode value of the Type I neurons (0.059 ± 0.006 vs. 0.111 ± 0.018, *p* = 0.011). As for the mode value of Type II neuron, the 6-OHDA lesioned rats showed no change in comparison to the control rats (0.078 ± 0.014 vs. 0.078 ± 0.007, *p* = 0.276). The mode value of Type III neurons was smaller in the lesioned rats than that in the control rats (0.035 ± 0.005 vs. 0.060 ± 0.008, *p* = 0.006).

For Type I, II, and III neurons, the asymmetry index (Figure 4D) slightly reduced after the 6-OHDA lesion. The value in the control and lesioned group was 0.304 ± 0.036 vs. 0.269 ± 0.030 (*p* = 0.389), 0.315 ± 0.046 vs. 0.246 ± 0.023, (*p* = 0.419), and 0.315 ± 0.024 vs. 0.265 ± 0.021 (*p* = 0.254), respectively.

Taken together, the mean firing rate in Type III neurons was higher than those in Type I and Type II neurons.

### Effects of the 6-OHDA-Lesion on the PPN LFP

**FIGURE 5:**
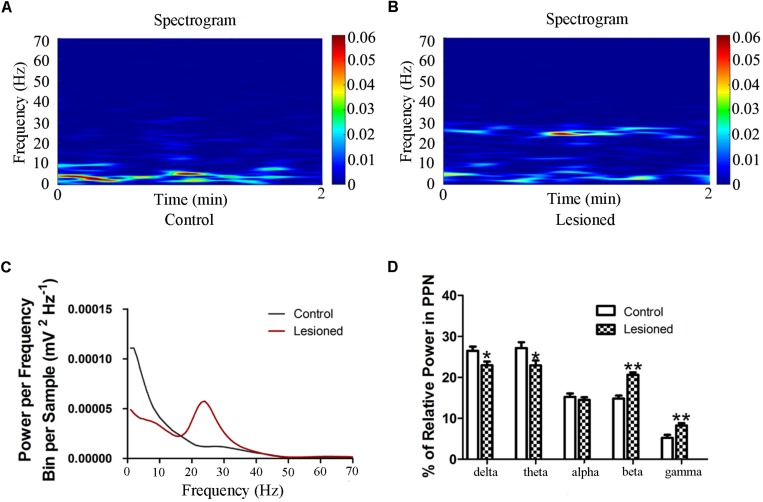
Power of PPN LFP activity in the 6-OHDA-lesioned rats during quiet wakefulness. Time-frequency plots of LFP spectral power in the PPN in control group **(A)** and lesioned group **(B)**. **(C)** Welch method shows a mean level of LFP activity in control and lesioned group **(D)** Bar graph represent relative LFP power in the PPN in control and lesioned group. (^∗^*p* < 0.05,^∗∗^*p* < 0.01).

We next investigated the PPN LFP between the 6-OHDA lesion group and the control group. Compared with that in the control group, the power spectrum of 6-OHDA lesion group was suppressed in the low-frequency, but enhanced in the high-frequency (F_(2.857, 217.094)_ = 20.454, *p* = 0.000) (Figure 5). To be specific, lesioned rats showed a lower relative power of delta and theta bands in the PPN compared to control rats (22.953 ± 0.881 vs. 26.454 ± 1.067, *p* = 0.016; 22.933 ± 1.187 vs. 27.146 ± 1.437, *p* = 0.030), whereas the power of beta (20.616 ± 0.607 vs. 14.823 ± 0.736, *p* = 0.000) and gamma (8.250 ± 0.581 vs. 5.232 ± 0.703, *p* = 0.002) bands was enhanced in the lesioned rats compared with those in the control rats (Figure 5D).

### Effects of the 6-OHDA-Lesion on PPN Spike-LFP Relationship

#### Type I (Cholinergic) Neuronal Spike and LFP Relationships

**FIGURE 6:**
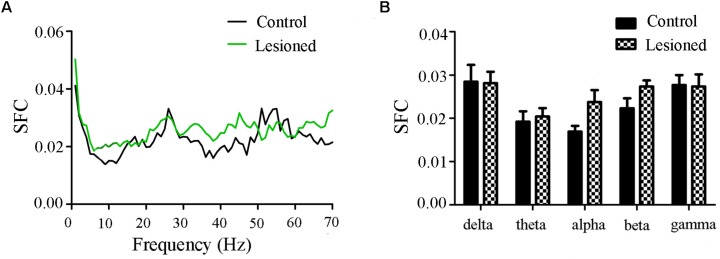
Firing of Type I neuron with respect to LFP activity in control and lesioned group. Comparison of the SFC across whole frequencies and different frequency bands in control and lesioned rats was shown in **(A,B)**.

Repeated measures ANOVA with Greenhose-Geisser correction determined that the SFC showed no difference between control and lesioned groups (F_(1.109,88.715)_ = 0.980, *p* = 0.334) (Figures 6A,B). This indicated the no synchronization between spikes and ongoing oscillation of whole frequency. We hypothesized that the change of spikes in cholinergic neuron arouse from the projection of other reciprocal regions. However, the statistical results showed that the average SFC value (across all type I neurons) in the alpha band was higher in the lesioned group than that in the control group (*p* = 0.043), while there was no change in other frequency bands (*p* = 0.235 for delta band, *p* = 0.179 for theta band, *p* = 0.063 for beta band, *p* = 0.307 for gamma band, respectively) (Figure 6B).

#### Type II (GABAergic) Neuronal Spike and LFP Relationships

**FIGURE 7:**
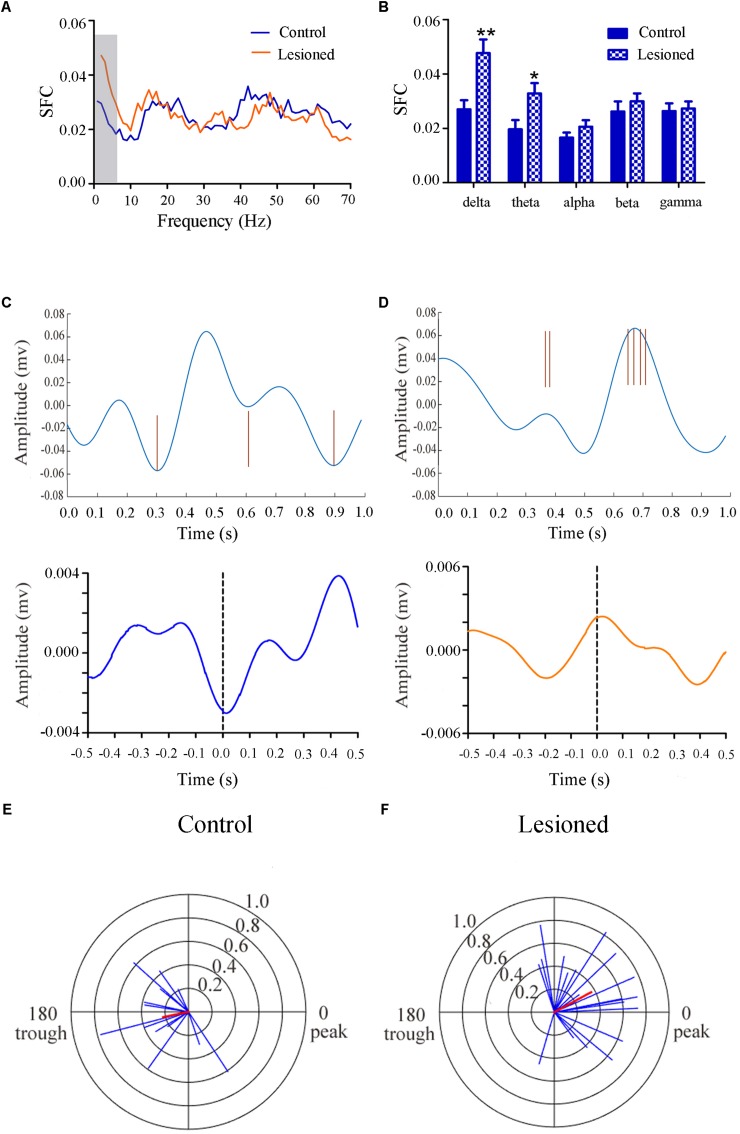
Firing of Type II neuron with respect to LFP activity in control and lesioned group. Comparison of the SFC across whole frequencies and different frequency bands in control and lesioned rats was shown in **(A,B)**. Single-unit examples illustrating spike occurrence at a consistent phase of delta oscillations in control and lesioned group was shown on the top of **(C,D)**. The brown line represents each spike count. Average STA across Type II neuron (bottom) denoted the phase preference of each spike in relation to delta band. The circular histogram for all Type II neurons (blue lines stands for each neuron, Rayleigh’s r, *p* < 0.05) were shown in control **(E)** and lesioned **(F)** rats. Mean vectors of preferred firing of subpopulation were shown as red lines radiating from the center. (^∗^*p* < 0.05,^∗∗^*p* < 0.01).

Repeated measures ANOVA with Greenhose-Geisser correction determined that the SFC differed greatly between control and lesioned groups (F_(3.049, 243.940)_ = 8.674, *p* = 0.000). The statistical results showed that the average SFC value (across all type II neurons) in the low frequency band was higher in the lesioned group than that in the control group (lesioned: 0.048 ± 0.004 and 0.033 ± 0.003 vs. control: 0.027 ± 0.006 for delta band and 0.020 ± 0.005, *p* = 0.008 and *p* = 0.028 for theta band), while there was no change in other frequency bands (*p* = 0.076 for alpha band, *p* = 0.120 for beta band, *p* = 0.669 for gamma band, respectively) (Figures 7A,B). This indicated the phase synchronization between spikes and low frequency oscillation. To explore straightforward distribution of spikes in relation to their oscillation, the 1–4 Hz frequency component of the signal together with a subset of the spikes were shown in Figures 7C,D (top). From Figures 7C,D (bottom), we observed that Type II neurons tended to fire close to the trough of delta-frequency oscillation in control rats, while mostly close to the peak in lesioned rats. Neurons had a range of phase preferences. Specifically, a substantial fraction of type II neurons produced spikes that were phase-locked to an oscillation in the 1–4 Hz range, causing a striking difference of phase angles value between both groups (lesioned: 28.40 ± 56.49° vs. control: 186.47 ± 51.45°, *p* = 0.001). In addition, all delta-locked neurons have an increased mean vector length value in the lesioned group (lesioned: 0.37 vs. control: 0.22, *p* = 0.001). The preferred delta phase distributions of phase-locked neurons in control and lesioned group were shown in Figures 7E,F. Together, these data indicated that the enhanced strength of spike phase-locking of GABAergic neuron spikes in relation to delta oscillations in lesioned rats.

#### Type III (Glutamatergic) Neuronal Spike and LFP Relationships

**FIGURE 8:**
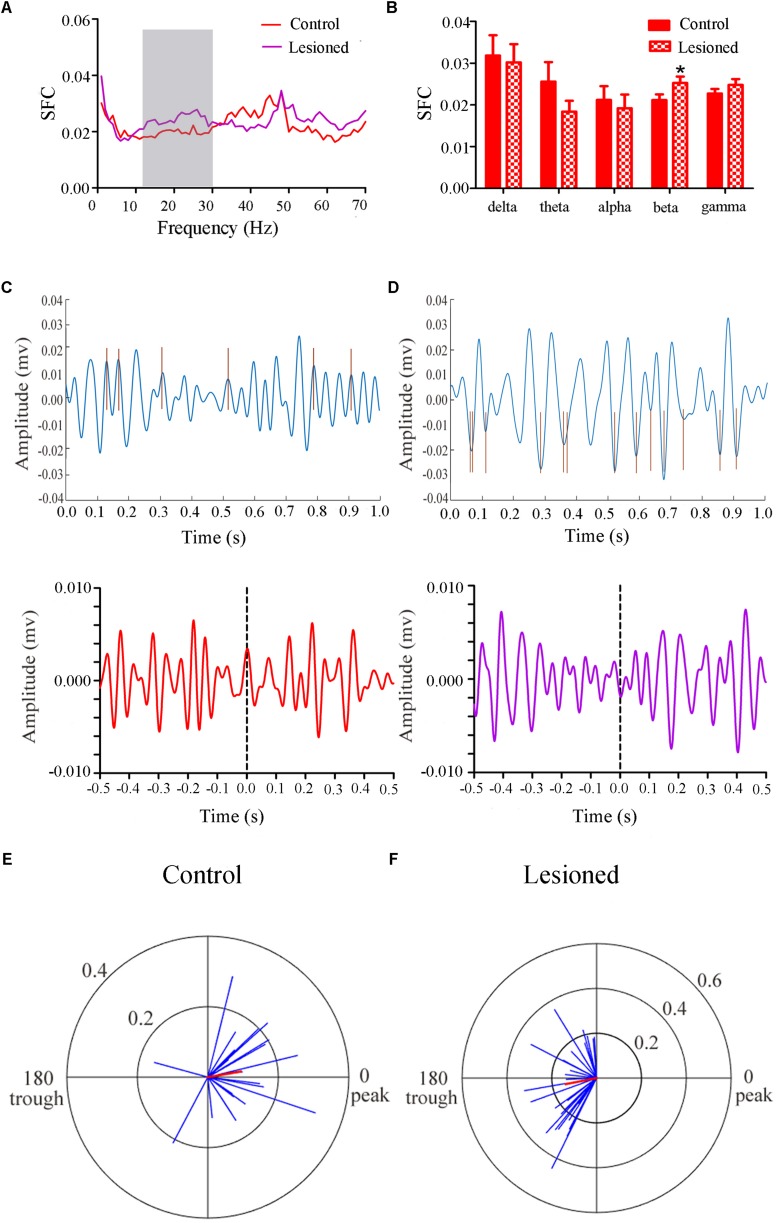
Firing of Type III neurons with respect to LFP activity in control and lesioned group. Comparison of the SFC across whole frequencies and different frequency bands in control and lesioned rats was shown in **(A,B)**. Single-unit examples illustrating spike occurrence at a consistent phase of beta oscillations in control and lesioned group was shown on the top of **(C,D)**. The brown line represents each spike count. Average STA across Type III neuron (bottom) denoted the phase preference of each spike in relation to beta band. The circular histogram for all Type III neurons (blue lines stands for each neuron, Rayleigh’s r, *p* < 0.05) were shown in control **(E)** and lesioned **(F)** rats. Mean vectors of preferred firing of subpopulation were shown as red lines radiating from the center (^∗^*p* < 0.05).

Repeated measures ANOVA with Greenhose-Geisser correction determined that the SFC differed greatly between control and lesioned groups (F_(2.493, 251.803)_ = 3.017, *p* = 0.039). The statistical results showed that the average SFC value (across all type III neurons) in the beta band (12–30 Hz) was higher in the lesioned group than that in the control group (lesioned: 0.026 ± 0.001 vs. control: 0.021 ± 0.002, *p* = 0.039), while there was no change in other frequency bands (*p* = 0.282 for delta band, *p* = 0.059 for theta band, *p* = 0.395 for alpha band, *p* = 0.156 for gamma band, respectively) (Figures 8A,B). This indicated the phase synchronization between spikes and low frequency oscillation. To explore straightforward distribution of spikes in relation to their oscillation, the 12–30 Hz frequency component of the signal together with a subset of the spikes were shown in Figures 8C,D (top). From Figures 8C,D (bottom), we observed that the Type III neuron tended to fire close to the peak of delta-frequency oscillation in control rats, while mostly was closer to the trough in lesioned rats. Neurons had a range of phase preferences. Specifically, a substantial fraction of type III neurons produced spikes that were phase-locked to an oscillation in the 12–30 Hz range, causing strikingly difference of phase angles value in both groups (lesioned: 181.50 ± 55.47° vs. control: 10.17 ± 41.44°, *p* = 0.003). In addition, all beta-locked neurons have increased mean vector length value in the lesioned group (lesioned: 0.026 ± 0.001 vs. control: 0.021 ± 0.002, *p* = 0.039). The preferred beta phase distributions of phase-locked neurons in control and lesioned group were shown in Figures 8E,F. Together, these data indicated that the enhanced strength of spike phase-locking of glutamatergic neuron spikes in relation to beta oscillations in lesioned rats.

Taken together, the electrophysiological data illustrated that the firing rate of Type I neuron increased in lesioned group, while Type II and Type III neuron in the lesioned group has higher firing rate and strengthen phase-locked relationship on delta and beta oscillations (12–30 Hz) compared with that in the control group, respectively.

### Estimation of PPN Area and Changes of the Neurons in the PPN Affected by PD

**FIGURE 9:**
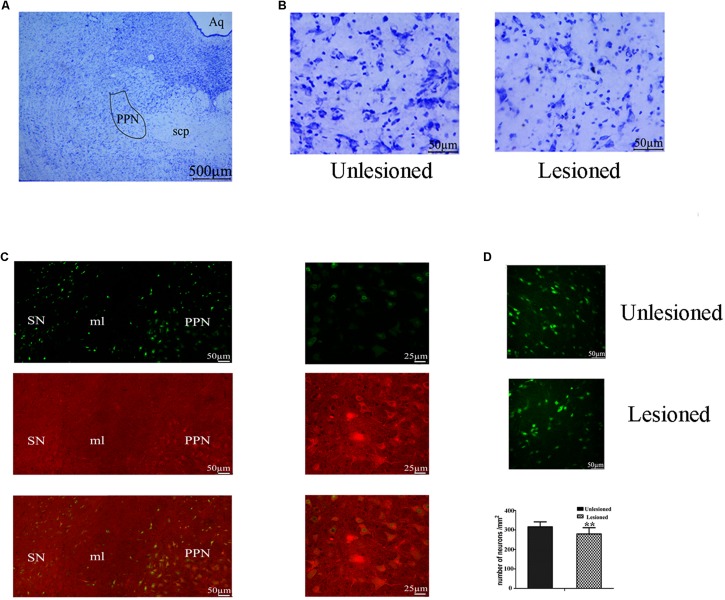
Change in the total neuron number of PPN between the non-lesioned side and the lesioned side. **(A)** Identification of PPN according to the Aq and scp (scp: superior cerebellar peduncle; Aq: aqueduct). Magnified areas stained by cresyl violet between the unlesioned side and the lesioned side showed in **(B)**. Scale bar = 500 μm; scale bar for magnification = 50 μm. **(C)** ChAT-positive neurons were observed to cross the boundary of the SN, as delimited by the medical leminiscus (ml) scale bar = 50 μm. High magnification showed the ChAT-positive neurons located in the PPN [**(C)** right] scale bar = 25 μm. Immunofluorescence quantitation of all neurons in the unlesioned side [**(D)** up] and the lesioned side [**(D)** middle]; scale bar = 50 μm. Stereological comparison of cell counts between the unlesioned side and the lesioned side [**(D)** bottom] (^∗∗^*p* < 0.01).

From Figure 9A, the PPN region is manually outlined in an irregular wedge-like shape and associated with the ascending limb of the superior cerebellar peduncles (scp), running parallel to the cerebral aqueduct (Aq). To delineate the boundary of PPN (Figure 9C), cholinergic populations provided a good guide. PPN started from the caudal end of the SN (representing the most rostral portion of the PPN) and extended caudally across the scp. In the view of the neural appearance (Figure 9B), the relatively large cells in this region were observed to be fusiform, triangular or multipolar, with thick primary dendrites, whereas medium-small sized cells were round or oval with thinner primary dendrites. After 6-OHDA lesion, neurons tended to have a smaller nucleus, larger nuclei, and diminished cell protrusions. From the perspective of quantity (Figure 9D), the number of neurons in the lesioned side decreased compared with that the non-lesioned side (279.56 ± 32.1 vs. 326.31 ± 25.25, *p* = 0.001).

### Morphological Change of Different Neurons After 6-OHDA Lesion

**FIGURE 10:**
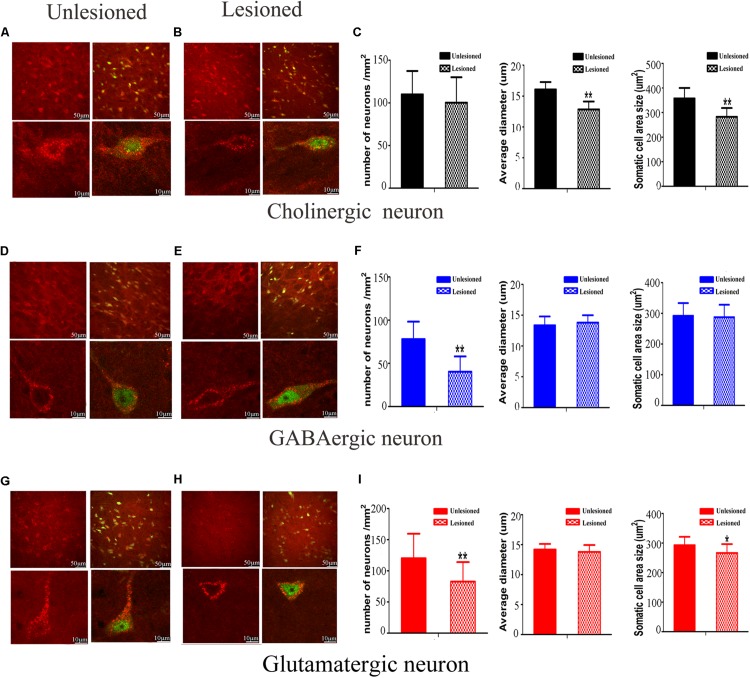
Photomicrographs of the double-immunofluorescence staining for cholinergic **(A,B)**, GABAergic **(D,E)**, and glutamatergic **(G,H)** neurons and the difference between the non-lesioned side and the lesioned side (scale bar = 50 μm). Three typical kinds of neurons were observed (scale bar = 10 μm). The quantitative analysis between the non-lesioned side and lesioned side were performed on cholinergic neuron **(C)**, GABAergic neuron **(F)** and glutamatergic neuron **(I)**, respectively. (^∗^*p* < 0.05, ^∗∗^*p* < 0.01).

We next compared the cellular morphology of each of the neuronal types in the lesioned side with the control side of the same rats. Cholinergic neurons were observed with medium-to-large cell body, in a polygonal, pyramidal or triangular shape (Figure 10A). These neurons became round or oval, with small soma on lesioned side (lesioned: 12.84 ± 1.28 μm vs. non-lesioned: 16.07 ± 1.20 μm, *p* = 0.002 in diameter) and (lesioned: 262.86 ± 35.76 μm^2^ vs. non-lesioned: 357.73 ± 42.44 μm^2^, *p* = 0.001 in area size) (Figures 10B,C). However, a similar neuronal density pattern was seen on both sides (lesioned: 100.31 ± 29.7 vs. non-lesioned: 109.94 ± 27.5, *p* = 0.302) (Figure 10C).

GABAergic neurons predominantly had a much smaller cell body, with a round or oval shape (Figure 10D). Following DA loss, statistical analysis didn’t detect big difference in diameter and cell body size (lesioned: 13.79 ± 1.44 μm vs. non-lesioned: 13.36 ± 1.20 μm, *p* = 0.369 in diameter) and (lesioned: 287.5 ± 40.52 μm^2^ vs. non-lesioned: 292.05 ± 41.56 μm^2^, *p* = 0.763 in area size) (Figures 10E,F). Surprisingly, a reduced density of GABAergic neurons, by approximately 49%, was seen on the lesioned side (lesioned: 40.31 ± 17.7 vs. non-lesioned: 78.00 ± 20.25, *p* = 0.001) (Figure 10F).

Glutamatergic neurons distributed principally on the posterior of PPN, in a fusiform shape (Figure 10G). After 6-OHDA lesion, the continuous diminishing of expression was seen at the polar end of their cell body (Figure 10H). The value of cell body area size appeared smaller (lesioned: 266.6 ± 29.76 μm^2^ vs. non-lesioned: 292.99 ± 28.24 μm^2^, *p* = 0.011) (Figure 10I). In terms of the numerical density, a dramatic decrease was observed on lesioned side (lesioned: 82.81 ± 31.20 vs. non-lesioned: 120.31 ± 39.25, *p* = 0.003).

Our analysis revealed that the 6-OHDA lesion results in notable morphological change in the PPN neurons. It exerts an effect not only on neuronal density but also on cell body area size for glutamatergic neuron, versus for cholinergic and GABAergic neurons only on either neuronal density or cell body area.

## Discussion

Our study aimed to characterize extracellular signal in terms of different subsets, phase relationship, and morphological pattern within the PPN region. We clustered the PPN neurons into three distinct types based on reliable electrophysiological features combined with positive expression of neurochemical markers. All three classified subtypes of PPN neurons in Parkinsonism showed obvious hyperactivities. Notably, the Type III (glutamatergic) neurons showed a dramatic increase in firing rate and fired spikes in relation to the trough of beta band oscillations after 6-OHDA lesion. Indeed, strong beta synchrony is a key pathophysiological symptom of parkinsonian circuits. Meanwhile, morphological damage (shrinkage of cell area and decreased number) was detected in glutamatergic neurons on lesioned side. This striking result implies that altered electrophysiological activity in glutamatergic neurons is most likely resulted from the dramatic morphological damage.

Most traditional work has been done to sort the neurons in PPN into two types (cholinergic and non-cholinergic neurons) (Zhang et al., 2008), with several findings even further dividing non-cholinergic neurons into GABAergic and glutamatergic neurons merely based on neurochemical property. However, their studies were limited to either anesthetized rats or rats at a sleep-wake switch (Boucetta and Jones, 2009; Sakai, 2012; Boucetta et al., 2014). Here, we recorded the electrophysiological features in wake condition. We did find three types of neurons in PPN were sorted based on waveform, duration and PCA, which further determined neurotransmitter phenotype with certainty by juxtacellular labeling and immunofluorescence identification. Accordingly, Type I neurons were regarded as cholinergic neurons (Zhang et al., 2008; Wang et al., 2016), Type II and Type III neuron were seen as GABAergic and glutamatergic neurons, respectively. In order to evaluate the separation capacity, L-ratio and ID were used to measure it (Petrantonakis and Panayiota, 2015).

Discharge activities of all three type- specific cells were enhanced after 6-OHDA lesion which could be explained by the fact that increasing excitatory projections from subthalamic nucleus (STN) play an important role in PD condition (Aravamuthan et al., 2008; Zhang et al., 2008). Considering spikes are only visible for cells laying within a few microns from electrodes, LFP was employed to measure average activity in the extracellular space. We found several bands of oscillatory activities had been changed, in particular, the beta (12–30 Hz) band modulation (Brown, 2006; Devergnas et al., 2014). Although LFP activity may be sensitive to the local information within the area, spike-LFP coherency is more effective in understanding neuronal connectivity and communication (Murer et al., 2002; Brazhnik et al., 2012). Nevertheless, studies that attribute beta band modulation to temporal coupling between specific-type neuron and accompanying LFP in PPN have not been reported yet.

Loss of GABAergic neuron was seen in a rat model of PD, which was also regarded as a feature of the PPN in PD patient (Pienaar et al., 2013). However, it is noteworthy that Type II (GABAergic) neurons spike-timing was predominantly trough-locked to delta band oscillations in the control rats, but were peak-locked in the lesioned rats (Aravamuthan et al., 2008). This is because GABAergic cells fired more frequently, and evoked greater membrane depolarization, thus easily locate at the trough of the oscillations, representing the periods of greatest negative potential in the extracellular space (Kajikawa and Schroeder, 2011).

An increase of synchronization of Type III (glutamatergic) neuron fired spikes in relation with LFP oscillation mainly in beta band was detected. The beta band abnormalities are believed to have anti-kinetic effects, and are likely to be responsible for some PD symptoms (Moran et al., 2011). This indicated that glutamatergic neurons are profoundly affected by the pathological changes. Accordingly, from morphological view, glutamatergic neurons in lesioned side had a distinct difference in cell appearance or quantification when compared to those in the non-lesioned side. Taken together, we hypothesized that abnormal activity of glutamatergic neuron was closely related to the imbalance of beta band oscillations, which predominately due to the damage of neuron.

The changes in the PPN spike timing relative to the PPN LFP oscillations following DA loss were observed in this study. Combined with the immunofluorescence, it pointed out that the increased synchronization is closely associated with the morphology, in particular, for non-cholinergic neurons. It is anticipated that such a view can be considered to be much better for the understanding of the direct or indirect contributions made by different cell types to the change of firing pattern in PPN. This study can facilitate our understanding of how to use the morphological view to explain the neuronal function in PD.

## Data Availability Statement

All datasets generated for this study are included in the manuscript/supplementary files.

## Ethics Statement

All animal welfare and surgery were conducted in accordance with the National Institutes of Health Guidelines for the Care and Use of Laboratory Animals.

## Author Contributions

All authors listed have made a substantial, direct and intellectual contribution to the work, and approved it for publication.

## Conflict of Interest

The authors declare that the research was conducted in the absence of any commercial or financial relationships that could be construed as a potential conflict of interest.

## Abbreviations

6-OHDA: 6-hydroxydopamine
ChAT: choline acetyltransferase
DA: dopamine
GABA: γ-aminobutyric acid
GAD67: GABAergic neurons with glutamate decarboxylase (67 kDa isoform)
MFB: medial forebrain bundle
PD: Parkinson’s disease
PPN: the pedunculopontine nucleus
SFC: Spike-field Coherence
SN: substantia nigra
STN: subthalamic nucleus
VGluT2: glutamatergic neurons with vesicular glutamate transporter 2.
